# Public Health Interventions for *Aedes* Control in the Time of Zikavirus– A Meta-Review on Effectiveness of Vector Control Strategies

**DOI:** 10.1371/journal.pntd.0005176

**Published:** 2016-12-07

**Authors:** Maha Bouzid, Julii Brainard, Lee Hooper, Paul R. Hunter

**Affiliations:** Norwich Medical School, University of East Anglia, Norwich, United Kingdom; Centers for Disease Control and Prevention, Puerto Rico, UNITED STATES

## Abstract

**Background:**

There is renewed interest in effective measures to control Zika and dengue vectors. A synthesis of published literature with a focus on the quality of evidence is warranted to determine the effectiveness of vector control strategies.

**Methodology:**

We conducted a meta-review assessing the effectiveness of any *Aedes* control measure. We searched Scopus and Medline for relevant reviews through to May 2016. Titles, abstracts and full texts were assessed independently for inclusion by two authors. Data extraction was performed in duplicate and validity of the evidence was assessed using GRADE criteria.

**Findings:**

13 systematic reviews that investigated the effect of control measures on entomological parameters or disease incidence were included. Biological controls seem to achieve better reduction of entomological indices than chemical controls, while educational campaigns can reduce breeding habitats. Integrated vector control strategies may not always increase effectiveness. The efficacy of any control programme is dependent on local settings, intervention type, resources and study duration, which may partly explain the varying degree of success between studies. Nevertheless, the quality of evidence was mostly low to very low due to poor reporting of study design, observational methodologies, heterogeneity, and indirect outcomes, thus hindering an evidence-based recommendation.

**Conclusions:**

The evidence for the effectiveness of *Aedes* control measures is mixed. Chemical control, which is commonly used, does not appear to be associated with sustainable reductions of mosquito populations over time. Indeed, by contributing to a false sense of security, chemical control may reduce the effectiveness of educational interventions aimed at encouraging local people to remove mosquito breeding sites. Better quality studies of the impact of vector control interventions on the incidence of human infections with Dengue or Zika are still needed.

## Author Summary

Various strategies for the control of mosquito-borne diseases exist and have been used for decades. The effectiveness of these control measures has been evaluated in several systematic reviews, however, their conclusions were contradicting. The current Zika outbreak in the Americas renewed the global health community’s interest in the control of *Aedes* transmitted diseases (dengue, yellow fever and chikungunya). We sought to provide an up to date systematic review about the effectiveness of chemical, biological, educational and integrated vector control strategies. In addition, we looked at recent primary studies that were not included in any systematic review as well as novel tools for mosquito control. This meta-review provides a comprehensive list of systematic reviews on the effect of vector control interventions on entomological parameters (most often indicators of vector density) or disease incidence. Biological control was found to achieve higher reduction of mosquito populations than chemical control. Educational campaigns are essential to reduce breeding sites and interrupt disease transmission. Integrated vector control strategies may not always increase effectiveness. The quality of the evidence was low to very low for most interventions. The effectiveness of any control strategy is setting- dependent.

## Introduction

The ongoing Zika virus outbreak in Central and South America which started in 2014 has attracted media attention and alarmed public health officials worldwide because of the high number of people affected, rapid transmission rate and association with immuno-neurological disorders (eg. Guillain-Barré syndrome) and newborn microcephaly [1–3]. It is feared that Zika virus will spread rapidly in the Americas as was the case for dengue and Chikungunya [2, 4]. Dengue fever, Zika, Chikungunya and yellow fever viruses are all transmitted by *Aedes aegypti* mosquitoes and associated with significant disease burden globally. While yellow fever is the only disease that has an effective vaccine, its incidence is increasing and it was stated that yellow fever is making a comeback due to the increasing number of naïve population following the scaling back of mass vaccination and changing sociodemographic conditions [5, 6]. *Aedes* is a genus of mosquitos which originated in Africa but are now found worldwide in tropical and subtropical zones. Establishment of *Aedes* mosquito, especially *A*. *aegypti*, has resulted in the epidemic spread of several arboviruses and linked to the current epidemic outbreak of Zika virus in South America [7]. The success of *A*. *aegypti* is linked to its opportunistic and high adaptability to the peridomestic environment exploiting any stagnant water as its breeding habitat [8]. Despite decades of *Aedes* mosquito control programmes, mosquito populations are widely established and abundant worldwide. Recognition of the link between Zika virus and newborn microcephaly in Brazil led to a concerted and renewed interest in *Aedes* control [7]. The World Health Organisation advice to control *Aedes* transmitted diseases is well implemented mosquito control measures that can effectively reduce disease transmission [8]. In order to assist the active implementation of *Aedes* control measures, we sought to provide a timely, up to date and evidence based synthesis of the literature.

We carried out a meta-review or “systematic review of systematic reviews” [9, 10], to assess and synthesise evidence from systematic reviews and meta-analyses. Meta-reviews allow evidence to be summarised on topics for which multiple systematic reviews have already been published [9, 11]. In addition, it may be possible to identify patterns of results not previously apparent, by taking into account a larger body of evidence than any individual systematic review captured. Meta-reviews provide a structured approach for exploring and explaining differences in systematic review conclusions, which may have resulted from variations in objectives, quality or other factors. This meta-review critically assessed systematic reviews that investigated the effectiveness of *Aedes* control interventions or protective measures against *Aedes* transmitted diseases.

## Methods

### Search methodology and inclusion criteria

In a previous meta-review investigating control strategies for a number of climate sensitive diseases, a broad search strategy retrieved five systematic reviews about dengue control [12]. For the current meta-review, the search was updated to retrieve recent systematic reviews on control of *Aedes* transmitted diseases (published between January 2011 and May 2016). Scopus and Medline Ovid databases were searched using the following search strategy: “(dengue OR chikungunya OR yellow fever OR Zika OR *Aedes*) AND (systematic review OR meta-analysis)”. This format was restricted to title, abstract and keyword fields. All systematic reviews reporting on the effectiveness of *Aedes* control measures were included. Reference lists from included reviews were screened for additional relevant reviews.

Titles, abstracts and full texts were assessed independently for inclusion by two authors. Data extraction was performed independently in duplicate using a standardised form and differences were resolved by discussion. Data extracted included type of intervention, main outcome measure, number of included studies, type of control group (pre-post, contemporary) and pooled effect size (when reported). The methodology and reporting were in accordance with the “Preferred Reporting Items for Systematic Reviews and Meta-Analyses” (PRISMA) [13] (S1 Checklist). The general approach adopted in this meta-review was based on the 2^nd^ edition of the World Health Organization’s Handbook for Guideline Development especially chapters 8 and 9 [14].

### Categorisation of vector control strategies

Vector control strategies were categorised as 1) Chemical controls (including insecticide and larvicide applications), 2) Biological controls (where a biological agent was used), 3) Educational campaigns (focused on training and awareness of the general public with the aim of reduction/ elimination of breeding sites) or 4) Integrated vector controls (comprising two or more individual control strategies) also known as Integrated Vector Management.

### Assessment of the quality of evidence using GRADE

The quality of the evidence was assessed using the GRADE score, recommended by the World Health Organisation [14], (http://www.gradeworkinggroup.org/) based on five criteria namely: risk of bias, imprecision, inconsistency, indirectness of evidence and publication bias [15]. Scores for each of these criteria were calculated and then combined for each intervention and by outcome measure. The overall score allowed to judge the quality of the evidence as very good, good, poor or very poor (for details of scoring see S1 Table).

## Results

**Fig 1:**
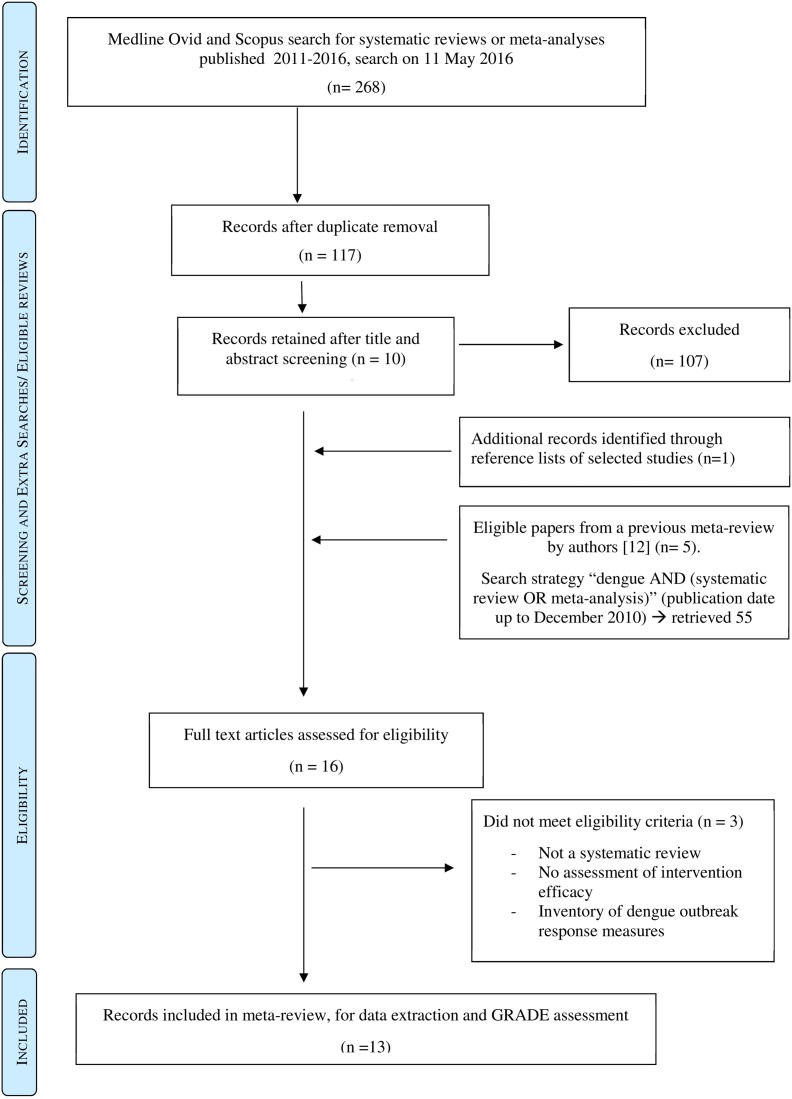
Flow diagram describing literature search, paper selection and inclusion/ exclusion process according to PRISMA guidelines.

165 articles published in 2011–2016 were retrieved from Scopus, 103 from Medline Ovid, with a total combined reduced to 177 after removal of duplicates. Following title and abstract screening, the full texts of 10 reviews were obtained and screened. Scanning of reference lists suggested one additional eligible systematic review. After full text analysis, three reviews were excluded. Five eligible systematic reviews from a previous meta-review [12] on control of climate-sensitive diseases were also included, leading to a final total of 13 included systematic reviews for data extraction and synthesis. The selection process is shown in Fig 1. The majority of systematic reviews dealt with dengue control. Many primary studies were included in multiple reviews.

### Overview of control measures in included systematic reviews

Control strategies were classified as chemical, biological, educational or integrated. For each included systematic review, control strategy, main outcome measure(s), number of included studies and effectiveness were recorded for each intervention type. Effectiveness was usually reported as pooled effect size for entomological indices or clinical outcomes. When pooled effects were not reported, descriptive analyses described by the authors were extracted instead. Table 1 summarises the characteristics of the included reviews.

**Table 1:** Effectiveness of *Aedes* control strategies reported in the systematic reviews included and evaluation of the quality of evidence

Intervention	Main outcome	Reference	Year of Publication	Number of included studies	Type of control group	Pooled effect size	GRADE summary score
**Chemical control (Insecticide spraying/ larvicide application)**							
Insecticide spraying (knockdown sprays)	Dengue incidence	[16]	2016	1	Cross sectional no control	Not applicable. OR 2.03 (95% CI 1.44–2.86)	Very low quality
Indoor insecticide spraying	Dengue incidence	[16]	2016	2	Not stated	Odds Ratio 0.67 (95% CI 0.22–2.11) (p=0.50)	Very low quality
Insecticide spraying and aerosols	Entomological index (House index)	[17]	2014	9 (out of 17)	Pre-post	Relative risk 0.90 (95% CI: 0.86–0.95) (10% reduction)	Very low quality
Insecticide spraying in peridomestic space	Dengue incidence	[18]	2010	1	Pre-post	Not applicable. The authors reported that new dengue cases dropped and only one case was detected 4 weeks after intervention	Very low quality
Insecticide spraying in peridomestic space	Entomological indices	[18]	2010	14	Pre-post	No pooled effect size was calculated because of heterogeneity of studies. 13 studies reported reduction in entomological indices, but these reductions were not sustained for long periods. The two remaining studies showed space spraying interventions to be ineffective	Very low quality
Outdoor insecticide spraying (adulticiding)	Entomological parameter (Breteau index)	[19]	2008	5 (out of 19)	Not stated, likely to be a mix of pre-post and contemporary controls	Relative effectiveness 0.24 (95% CI 0.05–1.19) (76% reduction)	Very low quality
Chemical control (insecticide spraying, chemical larvicides, insecticide-treated ovitraps)	Entomological parameters	[20]	2009	6 (out of 8)	Contemporary controls	Mean 27.2% (range 13.9–73.8%) (percent reduction using Mulla’s formula)	Very low quality
Temephos larvicide in water storage containers (single intervention)	Entomological parameters	[21]	2015	11	7 contemporary controls and 4 pre- post	No pooled effect size was calculated. All studies showed reduction in entomological indices	Very low quality
Insecticide treated curtains	Entomological indices	[16]	2016	2	Not stated	Mean difference -25.16 (95% CI -76.03–25.71) Breteau Index -10.58 (-32.22–11.05) House index	Very low quality
House screens	Dengue incidence	[16]	2016	3	Not stated	Odds Ratio 0.22 (95% CI 0.05–0.93)	Very low quality
Bed Nets	Dengue incidence	[16]	2016	2	1 No control and one not stated	Odds Ratio 0.91 (95% CI 0.49–1.67) (P=0.75)	Very low quality
Insecticide treated nets and curtains	Dengue positive serostatus	[17]	2014	4 (out of 17)	2 pre-post and 2 contemporary controls	0.30 (0.23–0.38) (70% reduction)	Low quality
Insecticide treated nets (ITNs)	Entomological parameters (pupae/person, indoor trap positivity, Breteau index)	[22]	2014	1 (out of 5)	Contemporary controls	Not applicable. 36% reduction in pupae per person and 77% reduction in indoor ovitrap positivity. However, ITNs were associated with a 56% increase in house index, 143% increase in container index, 60% increase in Breteau index and 20% increase in outdoor ovitrap positivity.	Low quality
Insecticide treated curtains	Entomological parameters (pupae/person, indoor trap positivity, Breteau index)	[22]	2014	3 (out of 5)	Contemporary controls	No pooled effect size was calculated. Reduction of entomological indices varied between studies and was much lower when follow up period exceeded 6 months	Low quality
Insecticide treated screens	Entomological indices: House Index (HI), Density Index (adults)	[22]	2014	1 (out of 5)	Pre-post	Not applicable. 100% reduction in both house and density indices. Both indices remained nil for the duration of the epidemic season (8 months post intervention), while seasonal peaks were observed in the control arm	Low quality
Insecticide treated screens	Clinical disease or infection (seroconversion)	[22]	2014	1 (out of 5)	Pre-post	Not applicable. Protective efficacy (PE) against IgM seropositivity 80% (95% CI: 53–92%, p < 0.001) (PE measures percentage reduction in risk of clinical disease or infection)	Low quality
Chemical insecticide (spraying and treated curtains)	Entomological indices	[23]	2015	5	Contemporary controls	Chi-square (w) = 57.27, pooled p-value (pw) < 0.0001	Very low quality
**Biological control**							
Copepods (crustaceans in water storage that eat larvae) used in community settings	Entomological parameters	[24]	2015	11	Contemporary controls	No pooled effect size was calculated. Descriptive results for each study were presented	Very low quality
Copepods (crustaceans in water storage that eat larvae) used in community settings	Positive dengue serology	[24]	2015	3 (of 11)	Contemporary controls	No pooled effect size was calculated. Reduction in seropositivity rates (IgM) were reported. However, no dengue cases were detected in both intervention and control communities for one study	Low quality
Larvivorous fish (single or multiple species) in water storage containers	Entomological parameters	[25]	2015	10 (out of 13)	2 pre-post and 8 contemporary controls	No pooled effect size was calculated. Descriptive results for each study were presented. Most studies reported reduction of entomological indices	Very low quality
Application of *Bacillus thuringiensis israelensis* (Bti) categorised as efficacy trials (mostly single application) and effectiveness trials (repeated application)	Entomological parameters (some studies calculated larval free period after intervention)	[26]	2013	14	Contemporary controls	No pooled effect size was calculated. Reduction of entomological indices varied between studies	Very low quality
Effectiveness of *Bacillus thuringiensis israelensis* (Bti)	Number of dengue cases	[26]	2013	1	Contemporary controls	Not applicable. One dengue case was reported in intervention area compared to 15 cases in control area	Very low quality
Biological controls (fish, crustaceans, aquatic insects, and bacteria based larvicide *Bacillus thuringiensis israelensis* (Bti))	Entomological indices	[23]	2015	5	Contemporary controls	Chi-square (w) = 72.51, pooled p-value (pw) < 0.0001	Very low quality
Biological intervention (copepods, Bti, turtles)	Entomological parameters	[20]	2009	5 (out of 8)	Contemporary controls	96.3% (range 75.1–100%) (percent reduction using Mulla’s formula)	Low quality
Biological control (larvivorous fish, copepods, predatory insect larvae)	Entomological parameter (Container index)	[19]	2008	9 (out of 10)	Not stated, likely to be a mix of pre-post and contemporary controls	Relative effectiveness 0.18 (95% CI 0.07–0.44) (82% reduction)	Very low quality
**Educational campaigns**							
Community based environmental management including use of water container covers	Dengue incidence	[16]	2016	1	Not stated	Not applicable. 0.22 (95% CI 0.15–0.32)	Low quality
Community based environmental modification (clean up, education, mobilisation and water covers)	Entomological indices	[16]	2016	2	Not stated	No pooled effect size was calculated.	Low quality
Preventive community based education and cleanliness campaigns	Ovitrap index	[17]	2014	3 (out of 17)	Pre-post	Relative risk 0.75 (95% CI: 0.62–0.91) (25% reduction)	Very low quality
Educational or behavioural interventions (screening, cleaning or disposal of water containers)	Entomological parameters	[20]	2009	5 (out of 8)	Contemporary controls	41.6% (range 4–87.6%) (percent reduction using Mulla’s formula)	Very low quality
Community based dengue control programmes (educational meetings and materials)	Entomological indices	[27]	2007	5 (out of 11)	4 pre-post and 1 contemporary control	No pooled effect size was calculated. All studies reported reduction in larval indices, though only two studies had statistically significant differences between intervention and control areas. One study did not measure entomological indices at baseline	Very low quality
**Integrated vector control measures (two or more control strategies)**							
Community based environmental modification (clean up, education, mobilisation and water covers) combined with larvicide application	Entomological indices	[16]	2016	1	Not stated	Not applicable. Rate Ratio 0.48 (95% CI 0.26–0.89) for Breteau Index	Very low quality
Temephos (larvicide) in water storage containers in combination with other measures (vector control and education campaigns)	Entomological parameters	[21]	2015	16	7 contemporary controls and 9 pre- post	No pooled effect size was calculated. Majority of studies combining temephos with chemical vector control showed reduction of entomological parameters but this was not sustained over time. The rest showed limited effectiveness of temephos	Very low quality
Larvivorous fish combined with other control measures (copepods, temephos, Bti, polystyrene beads, health education)	Entomological parameters	[25]	2015	3 (out of 13)	2 pre-post and 1 contemporary control	No pooled effect size was calculated. Descriptive results were presented. All studies reported reduction of entomological indices	Very low quality
Larvivorous fish alone or as part of integrated dengue control programme	Dengue cases	[25]	2015	2 (out of 13) one study for each category	2 pre-post	No pooled effect size was calculated. One study reported a dramatic decline and disappearance of dengue cases and the other study reported no dengue cases at all.	Very low quality
Integrated control (2 or more control strategies employed simultaneously including biological, chemical and mechanical (cleaning of containers and ovitraps) control as well as education campaigns)	Entomological parameters and number of dengue cases (3 studies)	[23]	2015	12	Contemporary controls	Chi-square (w) = 140.04, pooled p-value (pw) < 0.0001 (most effective strategy to control *A*. *aegypti*)	Very low quality
Community-based educational interventions (information materials and in house training) in combination or not with chemical and biological control (including indoor and outdoor insecticide spraying, chemical larviciding, covering, removal and clean-up of water containers, copepods)	Entomological indices	[28]	2011	22	6 pre-post and 16 contemporary	Relative effectiveness 0.25 (95% CI 0.17–0.37) calculated using the geometric mean of the different entomological indices reported in the included studies	Very low quality
Insecticide spraying in peridomestic space in combination with education campaign for elimination of breeding sites	Entomological indices	[18]	2010	1	Contemporary control	Not applicable. Houses that received both education and chemical sprays did not show significant reduction of entomological indices. Conversely, education campaigns alone achieved significant reduction of entomological indices. This suggests that chemical spraying could reduce the beneficial effect of educational interventions (attributed to false sense of security created by space spraying)	Very low quality
Educational interventions combined with either chemical or biological controls	Entomological parameters	[20]	2009	3 (out of 21)	Contemporary controls	No pooled effect size was calculated. Additionally, the authors allocated these studies to the relevant single intervention group and calculated percent reduction using Mulla’s formula seperately	Very low quality
Environmental management (removal of unused water vessels, covering of water containers, insecticide treated nets, curtains and screens)	Entomological parameters	[19]	2008	14	Not stated, likely to be a mix of pre-post and contemporary controls	Relative effectiveness 0.71 (95% CI 0.55–0.90) (Breteau index) (9 studies) 0.43 (95% CI 0.31–0.59) (Container index) (10 studies) 0.49 (95% CI 0.30–0.79) (House index) (10 studies)	Very low quality
Integrated vector management (environmental management combined with chemical vector control including outdoor and indoor spraying, bed nets, covering containers, water treatment with temephos)	Entomological parameters	[19]	2008	13	Not stated, likely to be a mix of pre-post and contemporary controls	Relative effectiveness 0.33 (95% CI 0.22–0.48) (Breteau index) (11 studies) 0.17 (95% CI 0.02–1.28) (Container index) (9 studies) 0.12 (95% CI 0.02–0.62) (88% reduction) (House index) (8 studies)	Very low quality
Integrated vector management (environmental management combined with biological vector control including covering containers, Bti, copepods, larvivorous fish, predatory larvae)	Entomological parameters	[19]	2008	5	Not stated, likely to be a mix of pre-post and contemporary controls	No pooled effect size was calculated as the authors stated that a minimum of five studies reporting on the same outcome measure are needed for meta-analysis. The five studies identified reported on different entomological indices	Very low quality
Community based educational dengue control programmes in combination with chemical larvicides	Entomological indices	[27]	2007	2 (out of 11)	1 pre-post and 1 contemporary control	No pooled effect size was calculated. Both studies showed significant reduction in entomological indices	Very low quality
Community based educational dengue control programmes in combination with chemical larvicides	Dengue incidence	[27]	2007	1 (out of 11)	Pre-post	Not applicable. Reduction of dengue incidence from 892 per 100000 to 685 per 100000	Very low quality
Community based educational dengue control programmes in combination with larvivorous fish and chemical larvicides	Entomological indices	[27]	2007	2 (out of 11)	Pre-post	No pooled effect size was calculated. Both studies showed reduction in entomological indices	Very low quality
Community based educational dengue control programmes in combination with copepods	Entomological indices	[27]	2007	1 (out of 11)	Pre-post	Not applicable. Reduction in entomological indices was reported	Very low quality
Community based educational dengue control programmes in combination with copepods	Dengue incidence	[27]	2007	1 (out of 11)	Pre-post	Not applicable. Significant reduction of dengue incidence from 1541 per 100000 to 0 per 100000	Very low quality

Reviews reporting on effectiveness of chemical control were most common (8/13), with 17 study arms (per type of intervention and outcome measure). All eight reviews [16–23] reported on the effects of chemical control on entomological indices and 4/8 [16–18, 22] on dengue incidence. Chemical control included insecticide spraying, insecticide treated curtains, nets and screens, and larvicide application (particularly temephos). Biological control was assessed in six reviews (8 study arms) and included copepods (crustaceans in water storage that eat mosquito larvae), larvivorous fish, *Bacillus thuringiensis israelensis* (Bti) bacterium, predatory insects and turtles. Copepods (n = 5) and Bti (n = 4) were the most widely reviewed biological agents. A single biological strategy was assessed in three reviews (5 study arms) and a combination of biological control strategies was assessed in three systematic reviews (3 study arms). For biological control, all six reviews [19, 20, 23–26] reported on entomological indices and two reviews [24, 26] also reported on dengue cases.

Four reviews (5 study arms) reported on educational campaigns (involving training, awareness raising and cleanliness incentives in households and/or for school children) as the only disease control measure. Educational campaigns aimed to reduce breeding sites by removing or covering water containers and elimination of water collection micro-habitats in the peridomestic environment. All four reviews [16, 17, 20, 27] reported on entomological indices and one [16] on dengue incidence. Integrated vector control strategies (details in Table 1) were assessed in 9/13 systematic reviews (16 study arms). Entomological indices were reported on in all nine reviews [16, 18–21, 23, 25, 27, 28] while only two reviews [25, 27] reported on dengue cases.

### Effectiveness of Chemical vector control

#### Insecticide spraying (adulticiding)

The most recent systematic review for dengue control was by Bowman and colleagues, who considered dengue incidence as their primary outcome measure [16]. Therefore, despite considering 19 primary studies, only a few studies were included for each intervention type. For insecticide spraying, only one observational study was included, which suggested a statistically significant negative effect (lower dengue incidence where spraying had not occurred), while for indoor insecticide spraying, two observational studies were included and the pooled Odds Ratio (OR) of 0.67 (95% CI 0.22–2.11) did not suggest any statistically significant effect. For both interventions, the evidence was of very low quality.

Das and colleagues included 17 primary studies (4 RCTs (randomised controlled trials) and 13 pre-post studies) [17]. Meta-analysis of nine pre-post studies of insecticide spraying and aerosols suggested a statistically significant 10% reduction in House Index (percentage of houses infested with larvae and/or pupae) (relative risk (RR) 0.90, 95% CI: 0.86–0.95), though effect on Breteau Index (number of containers with *Aedes* spp. larvae per 100 houses) in 2 RCTs was not statistically significant (both very low quality evidence). Ballenger-Browning and colleagues [20] reported an average 27% reduction in entomological indices after chemical control (insecticide, larvicides, ovitraps) in 3 RCTs and 3 clustered RCTs, but no meta-analysis was carried out and results were inconsistent between studies. Conversely, Erlanger and colleagues reported 76% reduction in Breteau Index (BI) after outdoor insecticide spraying based on five studies (Relative effectiveness 0.24 (95% CI 0.05–1.19), though this was not statistically significant [19]. Both systematic reviews provided very low quality evidence.

The review by Esu and colleagues focussed on effectiveness of peridomestic insecticide spraying, assessing entomological indices and dengue incidence [18]. They included 15 studies (including one pre-post study reporting on dengue incidence). Many studies were considered of poor quality and few took account of possible confounders, which is in accordance with our GRADE score suggesting very low quality evidence. No meta-analysis was reported because of the poor comparability of studies. The authors concluded that the evidence for the effectiveness of peridomestic space spraying was weak as reduction in entomological indices was not sustained over long periods of time [18].

#### Larviciding

George and colleagues reviewed efficacy of temephos in water storage containers (n = 11 studies) [21]. Four studies had pre-post design and seven had contemporary control groups. When temephos was used as an isolated intervention, all 11 studies reported a post-intervention reduction in the immature stages compared to their respective control group. It was observed that the treated sources were free of larvae for a variable period of time depending on the season of application, number of applications, dosage of temephos, procedure of control, and method of application. The authors did not pool results and concluded that there was insufficient evidence to conclude that temephos reduces dengue transmission. As study validity, publication bias and health outcomes were not reported, quality of evidence was very low.

#### Insecticide treated nets, curtains and screens

Bowman and colleagues assessed the effect of insecticide treated curtains on entomological indices based on 2 RCTs [16]. Statistically significant effects were not found for any of the four entomological indices assessed (for example, mean difference for Breteau Index was -25.16 (95% CI -76.03 to 25.71) with I^2^ 97% and for House index -10.58 (95% CI -32.22 to 11.05) I^2^ 97%)). Due to problems with allocation concealment, blinding and inconsistency, the quality of the evidence was very low. The same review evaluated the effect of home screens and bed nets on dengue incidence, though it was unclear whether these were insecticide treated [16]. For home screens, the pooled OR for dengue incidence was 0.22 (95% CI 0.05–0.93) based on 3 studies, but the evidence was of very low quality due to problems with confounding and selection bias in these observational studies. For bed nets a pooled OR of 0.91 was reported, though not statistically significant, which was very low quality evidence based on GRADE score.

Das and colleagues pooled data from four studies that assessed efficacy of domestic insecticide treated nets or curtains, and found a 70% reduction in dengue positive serotype status (RR: 0.30, 95%CI 0.23–0.38) [17]. Two studies were RCTs and two had a pre-post design. Due to lack of appropriate sequence generation and unclear blinding of assessors, the evidence was of low quality, despite the large effect size.

Wilson and colleagues reviewed the effect of insecticide treated nets (1 study), curtains (3 studies) and screens (1 study) on entomological indices and of insecticide treated screens on dengue seroconversion [22]. Four studies had contemporary control groups (RCTs) and one was pre-post design. Reductions in entomological indicators were reported in some individual studies, but no pooled effect size was calculated and results were inconsistent. The pre-post study reported 80% protective efficacy of insecticide treated house screens (95% CI 53 to 92%) against IgM seropositivity. The quality of evidence was low for both entomological outcomes and dengue seropositivity. Wilson and colleagues concluded that insecticide treated materials could reduce disease transmission, but reported that low *A*. *aegypti* mortality rates indicated significant insecticide resistance, which is likely to dramatically decrease the effectiveness of this type of control measure [22]. The authors highlighted that the study investigating dengue seropositivity used a non-randomised pre-post design and was deemed of low quality.

Lima and colleagues reviewed the effect of chemical control (insecticide spraying, growth regulators, insecticide treated items) based on 5 studies with contemporary control groups [23]. The pooled significance statistics (pw), which appear to equate to a standardised mean difference, suggested statistical significance. The quality of evidence was very low as methodology, validity and consistency of the included studies were not described.

### Effectiveness of biological control

#### Copepods

Lazaro and colleagues [24] reviewed the effectiveness of copepods introduced into water storage containers. All eleven studies were non-randomised interventions with contemporary comparator groups, providing very low quality evidence on entomological indicators (assessed in all studies) and low quality evidence on dengue seropositivity (assessed in three studies). No pooled effect size was calculated and results were presented descriptively. Copepods (*Mesocyclops* spp.) were effective for vector control in five community studies in Vietnam, including long-term control of larval and adult *A*. *aegypti* and dengue incidence [24]. However, this success was not replicated in studies conducted elsewhere. The authors attributed the Vietnam success to community participation, environmental and/or biological factors.

#### Larvivorous fish

Han and colleagues assessed the effect of larvivorous fish (single or multiple species) in water storage containers based on ten studies [25]. Two studies were pre-post comparisons and eight studies had contemporary comparators (though were not randomised), providing very low quality evidence. Results were presented descriptively and without quantitative pooling. Elimination of *Aedes* larvae was achieved in three studies. 9/10 studies reported a reduction in immature forms of dengue vector, two of which reported a continuous decline over 2 years. Reduction of adult mosquitoes was shown in only two studies.

#### *Bacillus thuringiensis israelensis* (Bti)

In Boyce and colleagues *Bacillus thuringiensis israelensis* (Bti), a bacterium that produces toxic proteins leading to high mortality among larvae after ingestion, was used as a dengue control measure [26]. 14 studies with contemporary control groups (4 RCTs, 10 clustered RCTs) reporting on entomological indices were included. 12 studies reported reductions in entomological indices, providing very low quality evidence. The authors reported that the two studies that did not show entomological reductions used environmental management or educational campaign in their control groups. Only one RCT reported on the effect of Bti as a targeted treatment of mosquito breeding sites on dengue cases. The treated area had one dengue case while 15 cases were recorded in the untreated area, however, this was considered very low quality evidence using the GRADE score. Given the large number of potential habitats and the impracticality of targeting them all, the authors concluded that the use of Bti as a single control measure may not achieve significant reductions in entomological indices and control dengue and other *Aedes* transmitted diseases [26].

#### Mixed biological interventions

Lima and colleagues considered a range of biological control measures (larvivorous fish, copepods, Bti and predatory insects) based on five studies, all with contemporary control groups [23]. The pooled significance statistic (pw), suggested statistical significance, but provided very low quality evidence. Ballenger-Browning and Elder also assessed several biological control measures (copepods, Bti and turtles) based on five studies that had contemporary control groups [20]. They reported 96.3% reduction in entomological indices (range 75.1–100%) based on Mulla’s formula, with a large effect size, though the evidence was of low quality. Erlanger and colleagues assessed a range of biological controls including larvivorous fish, predatory insect larvae and copepods [19]. The review was based on ten studies, nine of which were included in a pooled analysis, suggesting 82% reduction of container index (percentage of water containers positive for larvae/ pupae) (relative effectiveness 0.18, 95% CI 0.07 to 0.44), providing very low quality evidence of effectiveness, partly due to clear heterogeneity in study results. The one excluded study showed increased dengue risk in the intervention arm.

### Effectiveness of educational campaigns

Educational campaigns and community action interventions focus on educating and encouraging community members to take steps to reduce disease risk through environmental modification in order to reduce or eliminate mosquito’s breeding sites. While educational campaigns are rarely used as the sole control measure, four reviews assessed the effect of this control strategy on dengue transmission.

Bowman and colleagues included one RCT which assessed the effectiveness of community based environmental modification (including clean up, education, mobilisation and use of water container covers) on dengue incidence, finding a statistically significant reduction in dengue (OR 0.22, 95% CI 0.15 to 0.32), but providing only low quality evidence due to unclear allocation concealment, lack of blinding and lack of reproducibility. Evidence on entomological indices came from two studies, which appeared to lead to reductions in Breteau, House and Container Indices, however, the evidence was of low quality [16].

Das and colleagues assessed the effectiveness of preventive community based education and cleanliness campaigns based on three pre- post studies [17]. 25% reduction in ovitrap index (eggs found in traps per 100 houses) (RR 0.75, 95%CI 0.62–0.91) was reported, however, the quality of the evidence was very low. Ballenger-Browning and colleagues assessed the effect of educational or behavioural interventions (screening, cleaning or disposal of water containers) based on five studies with contemporary control groups [20]. They reported 41.6% mean reduction of entomological indices (range 4–87.6%), but this was very low quality evidence. Heintze and colleagues focussed on community-based control programmes (educational meetings and materials) based on five studies [27]. No pooled effect size was calculated as the authors found that most primary studies (all showing reductions in entomological indices) were of low quality, which was in accordance with the GRADE score.

### Effectiveness of integrated vector control measures

Integrated vector management refers to the simultaneous use of two or more control measures as detailed above. This type of control is favoured because it is thought to be more effective, which is reflected in the number of relevant systematic reviews (9/13).

George and colleagues reviewed the efficacy of temephos larvicide in water storage containers with other control measures (chemical or biological vector control, education campaigns) based on 16 studies [21]. Nine studies were pre-post design and seven were interventions with contemporary control groups (including 3 RCTs), providing very low quality evidence. No pooled effect size was calculated. Although 11 / 16 studies showed that temephos application together with other chemical vector control methods reduced entomological indices, this benefit was either not sustained over time or failed to reduce the immature stages (in 5 studies). The effectiveness of temephos depended on various factors including quality of delivery, water turnover rate, water type, organic debris, temperature and exposure to sunlight. In addition, long term success depended on political commitment and community participation. Limitations to temephos use and community effectiveness were identified as need for reapplication, cost, supplies, time consuming and laborious nature, high water turnover and temephos resistance as well as poor acceptability (due to unpleasant odour and taste) and limited local knowledge. Furthermore, it was reported that the use of temephos as part of an integrated strategy seemed to reduce implementation rate and effectiveness of source reduction and environmental management because of a false sense of security due to the belief that temephos application alone is sufficient to prevent dengue [21].

Han and colleagues assessed the effect of larvivorous fish in combination with other biological control measures and educational campaigns based on three studies (2 pre-post and 1 contemporary control group) [25]. All studies reported reductions in entomological indices though no pooled effect was calculated. The quality of evidence was very low. The same review assessed the effect of larvivorous fish alone or as part of integrated control on dengue cases based on two studies, providing very low quality evidence [25]. The first study found no dengue cases in any village since the start of the intervention, and the other study reported a decline from 6 cases pre-intervention to zero cases post-intervention, but the authors stated that this could not be attributed solely to the intervention.

Lima and colleagues investigated the effectiveness of integrated vector control combining biological, chemical and educational strategies based on 12 studies all with contemporary control groups [23]. The pooled significance statistic (pw) suggested statistical significance, but provided very low quality evidence.

Al-Muhandis and Hunter focussed on the role of community based educational interventions either alone or in combination with chemical or biological control (including indoor and outdoor insecticide spraying, larviciding, copepods, covering, removal and clean-up of water containers) [28]. This review included 22 studies (6 pre-post and 16 contemporary control groups), and reported a pooled relative effectiveness of 0.25 (95%CI 0.17–0.37) for entomological indices, with very low quality evidence. The authors reported that 61% of the heterogeneity in outcome measures could be explained by the type of control group and time from intervention to assessment. Studies using pre-post design substantially overestimated intervention effectiveness compared to studies using contemporary controls. It was noted that the effectiveness of educational interventions was maintained for about 18 months, and the authors observed that adding chemical or biological control to educational campaigns did not add value or increase effectiveness [28]. This finding was also reported by Esu and colleagues [18] who stated that houses that received both educational and chemical control did not achieve significant reduction of entomological indices, while houses that received educational campaigns alone achieved significant reduction. The authors concluded that chemical spraying may create a false sense of security and thus reduce the beneficial effect of educational campaigns.

Erlanger and colleagues assessed three types of integrated vector control strategies [19]. The first category focused on environmental management (removal of unused and covering of water containers) in combination with insecticide treated nets, curtains and screens and included 14 studies. The authors conducted pooled analyses, finding statistically significant reductions in three entomological indices, and providing very low quality evidence: Breteau Index (pooled BI, 0.71, 95% CI 0.55 to 0.90) based on 9 studies, Container Index (pooled CI, 0.43, 95% CI 0.31 to 0.59) and House Index (pooled HI, 0.49, 95% CI 0.30 to 0.79) (both based on 10 studies each). The second integrated control category was environmental management in combination with outdoor and indoor spraying as well as bed nets and larviciding. The pooled effect sizes suggested improvements in Breteau and House Indices, but not Container Index, though the evidence was of very low quality: (BI 0.33, 95%CI 0.22 to 0.48 based on 11 studies, CI 0.17, 95%CI 0.02 to 1.28 based on 9 studies and HI 0.12 95%CI 0.02 to 0.62 based on 8 studies). The third category was environmental management in combination with biological control, for which 5 primary studies were retrieved but no pooled effect was calculated as the studies reported on distinct entomological indices, providing very low quality evidence. Due to the consistent evidence of improvements in entomological indices, Erlanger and colleagues concluded that dengue vector control is effective in reducing vector populations [19]. However, their conclusion was not supported by the quality of evidence. The review did not report study methodology or assess study validity, study results were clearly heterogeneous, publication bias was unclear and no health outcomes were reported. The authors investigated intervention type as a source of heterogeneity and did not attempt to investigate whether excluding studies from pooling would bias their conclusions.

Heintze and colleagues focussed on community-based educational control programmes in combination with chemical larvicide and larvivorous fish or copepods based on 11 studies (2 RCTs, 6 pre-post studies and 3 interrupted time series) [27]. Each category was assessed separately and by outcome measure i.e. entomological indices and dengue incidence resulting in a very small number of primary studies per category. The authors reported that most studies were of low quality and concluded that the evidence of the effectiveness of community-based dengue control programmes is weak, which concurs with our GRADE score showing very low quality evidence for these interventions.

### Effectiveness of other control strategies

In addition to the widely used control strategies discussed above, Bowman and colleagues reviewed the effect of insect repellents (1 study), mosquito coils (2 studies) and mosquito traps (1 study) on dengue incidence [16]. The use of insect repellents and mosquito traps were not associated with a protective effect, while mosquito coils were significantly associated with an increased risk of dengue incidence (OR 1.44; 95% CI 1.09–1.91; p = 0.01). The quality of the evidence was very low.

## Discussion

Most included systematic reviews focussed on reducing entomological indicators. Undeniably, vector presence is pivotal for disease transmission, yet, there is no clear evidence of quantifiable association between vector density and disease transmission in particular whether reducing vector abundance actually leads to less disease [29]. This shortcoming was noticed by only a few systematic reviews’ authors. For example Heintze and colleagues stated “our findings suggest that although community-based control strategies in addition to or together with biological and chemical vector control tools are able to reduce classical *Aedes* larval indices, it is unknown whether this reduces dengue transmission” [27]. Therefore, evidence about entomological indices only was downgraded in our quality assessment for intervention impacts on disease incidence. Indeed, out of eight reviews that assessed the effect of vector control on disease outcomes [16–18, 22, 24–27], only two showed pooled statistically significant reduction in dengue incidence or positive serology [16, 17]. Future research on the effect of vector control strategies should utilise RCT methodology, have longer durations and report disease-related outcomes.

The strength of evidence for the effectiveness of any vector control intervention was uniformly low or very low. This means that while, in many cases, there was a suggestion of improvement, this was not scientifically rigorous, and we have little ability to compare effectiveness (or cost effectiveness) of different strategies. This was due to several reasons. Where dengue incidence was directly assessed, primary studies were generally observational, and intervention studies mostly assessed entomological outcomes (both observational studies and indirect outcomes downgraded the strength of evidence). The risk of bias in included studies was generally scored as very high due to problems with allocation concealment and blinding in intervention studies and lack of quality assessment or problems with confounding and dissimilarity of comparator groups at baseline for observational studies. In order to truly understand the effectiveness of dengue (and other vector-borne diseases) control interventions, we need high quality randomised controlled trials with adequate blinding, allocation concealment and sample size reporting on disease outcomes for long enough follow-up period.

Our review of the evidence was hampered by the quality of primary studies as well as some of the systematic reviews included. It was uncommon for systematic reviews to describe study methodology accurately or assess study validity appropriately or publication bias. These omissions have inevitably clouded our understanding of the levels of bias within the included primary studies [30]. It is possible that some evidence maybe of higher quality than assessed using the GRADE score, but in the absence of clear reporting, the quality of the evidence is downgraded.

Where reviews did not assess the underlying validity of the included studies, particularly study methodology (type of control group, randomisation, allocation concealment and blinding), the effectiveness of vector control strategies was more likely to be over-stated. Previous research established that using historical controls (pre-post studies) substantially over-estimated effectiveness compared to studies using contemporary control groups [28]. The use of historic controls is considered poor practice as most historical control groups are compromised [31, 32]. Many studies in the review by Erlanger and colleagues [19] had pre-post design, which may explain their conclusions that “dengue vector control is effective in reducing vector populations”, even though our assessment suggests very low quality evidence. However, other reviews surveying some of the same evidence were more cautious, such as Heintze and colleagues [27] who concluded “Evidence that community-based dengue control programmes … can enhance the effectiveness of dengue control programmes is weak”, Ballenger-Browning and Elder said “Little evidence exists to support the efficacy of mosquito abatement programs owing to poor study designs and lack of congruent entomologic indices” [20] and Esu and colleagues stated “Based on a comprehensive search of available peer reviewed literature, the effectiveness of peridomestic space spraying in reducing dengue transmission has not been conclusively demonstrated” [18].

While systematic reviews represent high quality evidence, we acknowledge that they might exclude relevant studies due to strict inclusion criteria and are limited to dated evidence i.e. by the time of publication, the recent literature could comprise relevant studies (potentially changing the body of evidence). Therefore, we attempted to provide a brief overview of latest relevant research that did not inform this meta-review, including novel vector control strategies that did not have ample body of evidence warranting consideration by systematic reviews’ authors. Further information is provided in S1 Text.

It is worth bearing in mind that effectiveness of any disease control intervention is closely related to the specific settings of the study area. For example, Lazaro and colleagues found that copepods were effective in studies carried out in Vietnam, including long-term control of larval and adult *A*. *aegypti* and dengue incidence [24]. However, this success was not replicated in studies conducted elsewhere (Costa Rica, Mexico, USA, Honduras, Laos). The authors attributed the success in Vietnam to community participation, environmental and/or biological factors. Tran and colleagues discussed social sustainability of copepods for dengue control in Vietnam, and reported that effectiveness varied between northern and central Vietnam (high sustainability) and south Vietnam (low sustainability) [33]. Limited knowledge and education, lack of government support, poor implementation and poor household monitoring were the main drivers of low sustainability and limited effectiveness [33]. Further investigations including qualitative research alongside RCTs may assist better understanding of crucial factors supporting or reducing the effectiveness of specific control interventions.

The World Health Organisation (WHO) recommends “integrated approaches that tackle all life stages of the mosquito and fully engage communities” for the control of Zika and other *Aedes* transmitted diseases [8]. This is in accordance with Heintze and colleagues that “multifaceted interventions are more effective than single interventions because a larger variety of barriers for change can be addressed” [27], which is also in line with social science theory [34]. However, two reviews found that adding additional chemical or biological interventions to educational campaigns did not increase efficacy [18, 28]. This was attributed to a false sense of security following insecticide spraying [18] and the belief that temephos alone is sufficient to control dengue transmission [21]. Therefore, our review suggests that the WHO is correct to reiterate that the most effective intervention to control disease and protect populations is the elimination of mosquito breeding sites [8], which would require sustained and ongoing education campaigns, resource allocation and good governance. This is particularly important considering the resilience of *A*. *aegypti* mosquitoes, with population numbers recovering and increasing shortly after vector control strategies have ceased [12]. While prevention of mosquito borne diseases has always focused on control of the mosquito vector, there is a debate about whether a rethink of control strategies is warranted. This is relevant considering the day biting pattern and low flight range (<100 m) of *Aedes* mosquitoes. These traits mean that vector control strategies should be focused not only on the peridomestic environment but also on day gathering places such as markets, schools, hospitals etc. and combined with better diagnosis and monitoring/ restriction of viremic persons’ movement, which has been found to be an important driver of dengue spatiotemporal clustering and disease spread [35]. In addition, relevant factors driving establishment of *Aedes* and spread of *Aedes* transmitted diseases need to be better understood and accounted for when designing control strategies such as international travel and trade, urbanisation, water storage practices, socioeconomic factors and global environmental change.

All the primary studies included in the systematic reviews were undertaken in low and middle income countries (LMICs). Caveats may need to apply if extrapolating public health research between LMICs and indeed to high income nations. The efficacy of any newly introduced vector control measure may depend on other control measures already in place [36]. Another knowledge gap identified here is the scarcity of data on cost effectiveness of vector control strategies in systematic reviews [27]. Bearing in mind that Health Economics is currently a major element in decision making processes, future studies should address this gap [37, 38]. This is particularly important considering the significant burden of dengue and other vector-borne diseases (including Zika and yellow fever) and the international commitment to improve global health and eradicate poverty related diseases with finite financial means.

## Conclusions

We identified thirteen systematic reviews assessing dengue or *Aedes* control strategies. Control strategies were categorised and the effect of interventions on entomological indices and disease incidence were recorded. Though some systematic reviews reported significant reduction of entomological indices, most reviews were considered to be of low to very low quality. This suggests that more high quality primary studies and well conducted systematic reviews that follow PRISMA reporting guidance and report on the quality of evidence [13] are still required for evidence based recommendations. The systematic reviews we assessed suggest that biological control achieves better and more sustainable reduction of entomological indices than chemical control. Educational campaigns and community engagement appear paramount in reducing breeding habitats in the peridomestic environment, although ongoing resources must be allocated to ensure educational interventions are maintained. Chemical control measures could be associated with a false sense of security leading to lesser community engagement with reduction/ elimination of breeding sites. Promising novel vector control strategies are being tested and would be a valuable addition to control mosquito borne diseases.

## Supporting Information

S1 Checklist PRISMA Checklist. (DOC)

S1 Table GRADE assessment of quality of evidence for public health interventions to reduce dengue and *Aedes* transmitted diseases. (DOC)

S1 Text Emerging evidence about *Aedes* control not included in systematic reviews. (DOCX)
