# Supplementary material for: Public Health Interventions for Aedes Control in the Time of Zikavirus– A Meta-Review on Effectiveness of Vector Control Strategies
Source: PLoS Negl Trop Dis. 2016 Dec 7;10(12):e0005176. doi: 10.1371/journal.pntd.0005176 (PMC5142773; doi:10.1371/journal.pntd.0005176)
Supplement: S1 Table — (DOC) [file pntd.0005176.s002.doc]

S1_Table: GRADE assessment of quality of evidence for public health interventions to reduce dengue and *Aedes* transmitted diseases

| **Intervention** | **Outcome** | **References** | **Risk of bias*** | **Inconsistency*** | **Indirectness of evidence*** | **Imprecision*** | **Publication bias**** | **Other issues***** | **GRADE score & quality of evidence** |
| --- | --- | --- | --- | --- | --- | --- | --- | --- | --- |
| **Chemical control – insecticide spraying (adulticiding)** | | |  |  |  |  |  |  |  |
| Insecticide spraying | Dengue incidence | Bowman et al, 2016 | Observational, 1 cross sectional study, confounding & selection bias problematic, -2 | Only one study, -1 | No, 0 | Not reported, but statistically significant negative effect, 0 | Unclear, but <10 studies, 0 | None apparent, 0 | -3, very low quality  BEST: -3, very low quality |
| Indoor insecticide spraying | Dengue incidence | Bowman et al, 2016 | Observational, case control & retrospective observational, confounding & selection bias problematic, -2 | I^2^ 78%, very different effects of the two studies, -1 | No, 0 | Not reported, not statistically significant, -1 | Unclear, but <10 studies, 0 | None apparent, 0 | -4, very low quality  BEST: -3, very low quality |
| Community based interventions for dengue – insecticide spraying and aerosols | House Index | Das et al, 2014 | Intervention,  9 before/after studies, -2 | Not reported, unclear, tight confidence intervals suggest consistency, 0 | Yes, no evidence on health outcomes, -1 | Not reported, but statistically significant effect, 0 | Unclear, but <10 studies, 0 | None apparent, 0 | -3, very low quality  BEST: -3, very low quality |
| Community based insecticide spraying | Mean Breteau Index | Das et al, 2014 | Intervention,  2 RCTs,  Validity unclear (reported but unclear which RCTs reported this outcome),  -2 | Unclear, -1 | Yes, no evidence on health outcomes, -1 | Not reported, no statistically significant effect, -1 | Unclear, but <10 studies, 0 | None apparent, 0 | -5, very low quality  BEST: -1, moderate quality |
| Peridomestic space spraying with insecticide | Dengue incidence (new dengue cases) | Esu et al, 2010 | Intervention  1 before-after study, validity unclear, -2 (best -1) | Only one study assessed outcome, -1 | No, 0 | Number of events and participants unclear, -2 | Unclear, but <10 studies, 0 | None apparent, 0 | -5, very low quality  BEST: -2 low quality |
|  |  |  |  |  |  |  |  |  |  |
| Outdoor insecticide spraying | Entomological parameters | Erlanger et al, 2008 | Intervention  5 studies,  Validity unclear, -2 | Results of the 5 studies clearly heterogeneous, -1 | Yes, no evidence on health outcomes, -1 | ~1700 houses, 0 | Undetected, 0 | None apparent, 0 | -4, very low quality  BEST: -2, low quality |
| Insecticide spraying | Entomological parameters | Ballenger-Browning and Elder, 2009 | Intervention  3 RCTs & 3 CCTs, validity unclear, -2 | Results of the 6 studies not consistent, -1 | Yes, no evidence on health outcomes, -1 | 2112 houses, 0 | Unclear, but <10 studies, 0 | None apparent, 0 | -4, very low quality  BEST: -1 moderate quality |
| **Chemical control - larviciding** | | |  |  |  |  |  |  |  |
| Temephos in water-storage containers | Entomological indices | George et al, 2015 | 7 CCTs, 4 before/after,  Validity unclear, -2 | Results of the studies not consistent but different levels of effectiveness explored, 0 | Yes, no evidence on health outcomes, -1 | Sample size unclear but large, 0 | Unclear, -1 | None apparent, 0 | -4, very low quality  BEST: -1, moderate quality |
| **Chemical control – insecticide treated nets, curtains & screens** | | | |  |  |  |  |  |  |
| Insecticide treated curtains | Entomological indices | Bowman et al, 2016 | Intervention, 1 or 2 RCTs (depending on outcome), allocation concealment & blinding all at unclear or high risk of bias, -2 | Inconsistent results where there were 2 RCTs for a single outcome (I^2^ 97% in both cases), -1 | Yes, entomological outcomes, -1 | Not reported, not statistically significant, -1 | Unclear, but <10 studies, 0 | None apparent, 0 | -5, very low quality  BEST: -4, very low quality |
| House screens (treated or untreated) | Dengue incidence | Bowman et al, 2016 | Observational, 2 case control & 1 cross sectional, confounding & selection bias problematic, -2 | I^2^ 95% but all 3 studies showed statistically significant reductions in dengue, 0 | No, 0 | Not reported, but statistically significant effect, 0 | Unclear, but <10 studies, 0 | Large effect size (OR<0.5 for all 3 studies), +1 | -1, very low quality  BEST: -1, very low quality |
| Bed nets | Dengue incidence | Bowman et al, 2016 | Observational, 1 case control & 1 cross sectional, confounding & selection bias problematic, -2 | I^2^ 0%, 0 | No, 0 | Not reported, not statistically significant, -1 | Unclear, but <10 studies, 0 | None apparent, 0 | -3, very low quality  BEST: -2, very low quality |
| Insecticide treated bed nets and curtains | Dengue Positive serostatus | Das et al, 2014 | Intervention,  2 RCTs plus 2 before/after,  For RCTs sequence generation not done, blinding of assessors unclear, -2 | I^2^ >50%, though results of RCTs similar to those of pre-post studies, -1 | No, seropositive status, 0 | Not reported, but statistically significant effect, 0 | Unclear, but <10 studies, 0 | Large effect size (RR 0.3), +1 | -2, low quality  BEST: -2, low quality |
| Insecticide-treated nets, curtains and screening (assessed separately) | Entomological parameters | Wilson et al, 2014 | Intervention,  4 RCTs, 1 pre-post. RCTs generally at low risk of bias (pre-post below), 0 | Results not consistent, -1 | Yes, entomological outcomes, -1 | Sample size unclear, but over 7000 houses, 0 | Unclear, but <10 studies, 0 | None apparent, 0 | -2, low quality  BEST: -2 low quality |
| Insecticide-treated nets, curtains and screening | Dengue transmission | Wilson et al, 2014 | Intervention,  1 pre-post study, no allocation concealment or blinding, -2 | Only one study, -1 | No, 0 | 2 villages, ~1000 houses, no imprecision, 0 | Unclear, but <10 studies, 0 | Protective efficacy 80%, large effect size, 1 | -2, low quality  BEST: -2, low quality |
| Chemical agents | Entomological indices | Lima et al, 2015 | Methodology unclear, validity unclear, -2 | Consistency unclear, -1 | Yes, limited data on health outcomes, -1 | Sample size unclear but large, 0 | Unclear, -1 | None apparent, 0 | -5, very low quality  BEST: -1 moderate quality |
| **Biological control - copepods** | | |  |  |  |  |  |  |  |
| Cyclopoid copepods (Mesocyclops spp) | Larval rates, mosquito rates | Lazaro et al, 2015 | Intervention,  11 CCTs, validity unclear, -2 | Results not consistent and heterogeneity explored, 0 | Yes, entomological outcomes, -1 | Sample size unclear but large, 0 | Unclear, -1 | None apparent, 0 | -4, very low quality  BEST: -1 moderate quality |
| Cyclopoid copepods (Mesocyclops spp) | Dengue transmission | Lazaro et al, 2015 | Intervention,  3 CCTs, validity unclear, -2 (Best -1) | Results consistent, 0 | No, evidence on health outcomes, 0 | Sample size unclear but large, 0 | Unclear, but <10 studies, 0 | None apparent, 0 | -2, low quality  BEST: -1 moderate quality |
| **Biological control – Larvivorous fish** | | |  |  |  |  |  |  |  |
| Larvivorous fish | Larval counts, breeding sites, mosquito density, container index, infested containers, infestation rate and incidence rate. | Han et al, 2015 | Intervention,  10 non-RCTs (exact design unclear),  Validity unclear, -2 | Results not consistent and heterogeneity explored, 0 | Yes, -1 | Sample size unclear but large, 0 | Unclear, -1 | None apparent, 0 | -4, very low quality  BEST: -1 moderate quality |
| Larvivorous fish | Dengue cases | Han et al, 2015 | Intervention,  1 non-RCT (exact design unclear),  Validity unclear, -2 | Consistent, no dengue cases reported in either study area, 0 | No, dengue cases reported, 0 | Sample size unclear but large, 0 | Unclear, but <10 studies, 0 | None apparent, 0 | -2, low quality  BEST: 0, high quality |
| **Biological control – bacillus thurigiensis israelensis (Bti)** | | | |  |  |  |  |  |  |
| Bacillus thuringiensis israelensis (Bti) as a single agent | Entomological (immature) indices | Boyce et al, 2013 | Intervention  4 RCTs, 10 CCTs,  Validity unclear, -2 | Results not consistent and heterogeneity explored, 0 | Yes, entomological indices, -1 | Sample size unclear as containers were the unit studied, not humans, but large number of sites, 0 | Unclear, -1 | None apparent, 0 | -4, very low quality  BEST: -1 moderate quality |
| Bacillus thuringiensis israelensis (Bti) as a single agent | Dengue cases | Boyce et al, 2013 | Intervention  1 RCT,  Validity unclear, -2 | Only one study, -1 | No, dengue cases, 0 | Sample size unclear as containers were the unit studied, not humans, but large number of sites, 0 | Unclear, but <10 studies, 0 | None apparent, 0 | -3, very low quality  BEST: -1, moderate quality |
| **Biological control - mixed** | | | |  |  |  |  |  |  |
| Biological agents | Entomological indices | Lima et al, 2015 | Methodology unclear, validity unclear, -2 | Consistency unclear, -1 | Yes, limited data on health outcomes, -1 | Sample size unclear but large, 0 | Unclear, -1 | None apparent, 0 | -5, very low quality  BEST: -1 moderate quality |
| Biological intervention | Entomological parameters | Ballenger-Browning and Elder, 2009 | Intervention  5 CTs, validity unclear, -2 | Results of the 5 studies consistent, 0 | Yes, no evidence on health outcomes, -1 | 66 containers & 12 communes & 553 houses, 0 | Unclear, but <10 studies, 0 | Large effect size (75-100% reduction), +1 | -2, low quality  BEST: 0 high quality |
| Biological control of the vector (e.g. larvivorous fish, predatory insect larvae, copepods) | Entomological parameters | Erlanger et al, 2008 | Intervention  9 studies, validity unclear, -2 | Results of the 9 studies clearly heterogeneous, -1 | Yes, no evidence on health outcomes, -1 | Unclear but >2000 houses, 0 | Strongly suspected, -1 | Large effect size, +1 | -4, very low quality  BEST: -2 low quality |
| **Educational campaigns** | | |  |  |  |  |  |  |  |
| Community based environmental management including covers to water containers | Dengue incidence | Bowman et al, 2016 | Intervention, 1 RCT, allocation concealment unclear, blinding high risk, -2 | Only one study, -1 | No, 0 | Not reported, but statistically significant effect, 0 | Unclear, but <10 studies, 0 | Large effect size (OR <0.5), +1 | -2, low quality  BEST: -2 low quality |
| Community based environmental modification (clean up, education, mobilisation and water covers) | Entomological indices | Bowman et al, 2016 | Intervention, 1 RCT, low risk from allocation concealment, high risk from blinding, -1 | Consistent, all outcomes favoured intervention, 0 | Yes, entomological outcomes, -1 | Not reported, but statistically significant effect for each outcome, 0 | Unclear, but <10 studies, 0 | None apparent, 0 | -2, low quality  BEST: -2, low quality |
| Community based education and cleanliness campaigns | Ovitrap index | Das et al, 2014 | Intervention,  3 before/after studies, -2 | Unclear, -1 | Yes, no evidence on health outcomes, -1 | Not reported, but statistically significant effect, 0 | Unclear, but <10 studies, 0 | None apparent, 0 | -4, very low quality  BEST: -3, very low quality |
| Educational or behavioural interventions | Entomological parameters | Ballenger-Browning and Elder, 2009 | Intervention  5 trials, validity unclear, -2 | Results of the studies not consistent though all suggested protection, -1 | Yes, no evidence on health outcomes, -1 | 187 houses + 13 4-block communities, 15 neighbourhoods, 0 | Unclear, but <10 studies, 0 | None apparent, 0 | -4, very low quality  BEST: -1 moderate quality |
| Community based dengue control programmes (education alone) | Entomological indices, detection of larval stages | Heintze et al, 2007 | Intervention  1 RCTs, 3 before/after,  1 interrupted time series, validity unclear, -2 | Results of the studies not consistent, -1 | Yes, entomological indices, -1 | >100,000 people, 0 | Unclear, -1 | None apparent, 0 | -5, very low quality  BEST: -1 moderate quality |
| **Integrated control measures** | | |  |  |  |  |  |  |  |
| Community based environmental modification (clean up, education, mobilisation and water covers) combined with larvicide application | Entomological indices | Bowman et al, 2016 | Intervention, 1 RCT for each outcome, unclear risk from allocation concealment, high risk from blinding, -2 | Consistent, all outcomes favoured intervention, 0 | Yes, entomological outcomes, -1 | Not reported, but statistically significant effect for each outcome, 0 | Unclear, but <10 studies, 0 | None apparent, 0 | -3, very low quality  BEST: -3, very low quality |
| Temephos in water-storage containers plus other biological, chemical, and/or educational interventions | Entomological indices | George et al, 2015 | 3 RCTs, 4 CCTs, 9 before/after,  Validity unclear, -2 | Results of the studies not consistent, -1 | Yes, no evidence on health outcomes, -1 | Sample size unclear but large, 0 | Unclear, -1 | None apparent, 0 | -5, very low quality  BEST: -1, moderate quality |
| Larvivorous fish combined with other control measures | Larval counts, breeding sites, mosquito density, container index, infested containers, infestation rate and incidence rate. | Han et al, 2015 | Intervention,  1 CCT, 2 pre-post studies,  Validity unclear, -2 | Results not consistent and heterogeneity explored, 0 | Yes, -1 | Sample size unclear but large, 0 | Unclear, -1 | None apparent, 0 | -4, very low quality  BEST: -1 moderate quality |
| Larvivorous fish as part of an integrated dengue control strategy | Dengue cases | Han et al, 2015 | Intervention,  1 pre-post study, Validity unclear, -2 | Only one study, -1 | No, dengue cases reported, 0 | Sample size unclear but large, 0 | Unclear, but <10 studies, 0 | None apparent, 0 | -3, very low quality  BEST: 0, high quality |
| Integrated interventions | Entomological parameters and number of dengue cases | Lima et al, 2015 | Methodology unclear, validity unclear, -2 | Consistency unclear, -1 | Yes, limited data on health outcomes, -1 | Sample size unclear but large, 0 | Unclear, -1 | None apparent, 0 | -5, very low quality  BEST: -1 moderate quality |
| Community-based educational interventions alone or combined with chemical or biological control | Entomological indices | Al-Muhandis and Hunter, 2011 | Intervention  22 with concurrent or historical controls, validity unclear, analysed effects of other methodological issues, -2 | Results not consistent and heterogeneity explored, 0 | Yes, no evidence on health outcomes, -1 | Sample size unclear but large, 0 | Unclear, -1 | None apparent, 0 | -4, very low quality  BEST: -1 moderate quality |
| Peridomestic space spraying with insecticide in combination with education campaigns for elimination of breeding sites | Entomological indices | Esu et al, 2010 | Intervention  1 RCT, 1 CCT, 13 before/after studies, validity unclear, -2 | 13/15 studies suggested protection, not further reported, -1 | Yes, no evidence on health outcomes, -1 | 187 houses, -1 | Unclear, -1 | None apparent, 0 | -6, very low quality  BEST: -3 very low quality |
| Educational interventions combined with either chemical or biological controls | Entomological parameters | Ballenger-Browning and Elder, 2009 | Intervention, 3 studies, validity unclear, -2 | Consistency unclear, -1 | Yes, no evidence on health outcomes, -1 | Not reported, effect unclear, -1 | Unclear, but <10 studies, 0 | None apparent, 0 | -5, very low quality  BEST: -1 moderate quality |
| Environmental management (e.g. removal of unused water vessels and covering of water containers, insecticide treated nets, curtains and screens) | Entomological parameters | Erlanger et al, 2008 | Intervention  9 studies, validity unclear, -2 | Results of the 9 studies clearly heterogeneous, -1 | Yes, no evidence on health outcomes, -1 | Unclear but >2500 houses, 0 | Strongly suspected, -1 | None apparent, 0 | -5, very low quality  BEST: -3 very low quality |
| Integrated vector management (environmental management combined with vector control) | Entomological parameters | Erlanger et al, 2008 | Unclear  18 studies, validity unclear, -2 | Results of the 18 studies clearly heterogeneous, -1 | Yes, no evidence on health outcomes, -1 | Sample size unclear but large, 0 | Unclear, -1 | Large effect size (all summary Res <0.5), +1 | -4, very low quality  BEST: -1 very low quality |
| Community based dengue control programmes (education and/or insecticide spraying and/or biological control) | Entomological indices, detection of larval stages | Heintze et al, 2007 | Intervention  2 RCTs, 6 before/after,  3 interrupted time series, validity unclear, -2 | Results of the 2 studies not consistent, -1 | Yes, entomological indices, -1 | >100,000 people, 0 | Unclear, -1 | None apparent, 0 | -5, very low quality  BEST: -1 moderate quality |
| Community based dengue control programmes (education and/or insecticide spraying and/or biological control) | Confirmed dengue cases | Heintze et al, 2007 | Intervention  1 before/after,  1 interrupted time series, validity low, -2 | Effect sizes and statistical significance not reported, unclear, -1 | No, 0 | Sample size unclear but large, 0 | Unclear, but <10 studies, 0 | None apparent, 0 | -3, very low quality  BEST: -2, low quality |
| **Other strategies** | | |  |  |  |  |  |  |  |
| Insect repellents | Dengue incidence | Bowman et al, 2016 | Observational, 1 cross sectional study, confounding & selection bias problematic, -2 | Only one study, -1 | No, 0 | Not reported, not statistically significant, -1 | Unclear, but <10 studies, 0 | None apparent, 0 | -4, very low quality  BEST: -3, very low quality |
| Mosquito coils | Dengue incidence | Bowman et al, 2016 | Observational, 1 case control & 1 cross sectional, confounding & selection bias problematic, -2 | I^2^ 0%, 0 | No, 0 | Not reported, but statistically significant negative effect, 0 | Unclear, but <10 studies, 0 | None apparent, 0 | -2, very low quality  BEST: -2, very low quality |
| Mosquito traps | Dengue incidence | Bowman et al, 2016 | Observational, 1 case control, confounding & selection bias problematic, -2 | Only one study, -1 | No, 0 | Not reported, not statistically significant, -1 | Unclear, but <10 studies, 0 | None apparent, 0 | -4, very low quality  BEST: -3, very low quality |

*Each assessed on the scale “no” (score 0), “serious” (score -1) or “very serious” (score -2). Where scores appear in red this was because the downgrading was due to lack of reporting in the systematic review (and may or may not indicate that the underlying research base was at risk of bias). The basis of assessment was:

- Risk of bias: Allocation concealment (AC), lack of blinding, incomplete accounting for fate of participants and selective outcome reporting – where none were assessed “validity unclear” was stated
- Inconsistency: Unexplained heterogeneity of results
- Indirectness of evidence: Presence of an indirect comparison or indirect evidence (studies did not directly address the question)
- Imprecision: For dichotomous outcomes imprecision was indicated by <300 total events, for continuous outcomes by total population size <400

**Publication bias was assessed as “undetected” (score 0) or “strongly suspected” (score -1) on the basis that it has been assessed and no evidence of bias has been found

***Other issues would upgrade the evidence, and include: large effect size (RR>2 or <0.5 score +1, RR>5 or <0.2 score +2), confounders working against bias (score +1), and/or presence of a dose response (score +1)

**GRADE summary score was the addition of the previous scores.**

For evidence based on **intervention** studies (white rows): Score ≥0 equated to “high quality”, -1 “moderate quality”, -2 “low quality” and ≤-3 “very low quality”.

For evidence based on **observational** studies (blue or yellow rows): Score ≥+2 equated to “high quality”, +1 “moderate quality”, 0 “low quality” and ≤-1 “very low quality”.

The “BEST” GRADE score was the best possible score of the underlying evidence (assuming that characteristics not reported in reviews were all ideal), the GRADE score removing where the score was downgraded due to the review being unclear.

**Grading methodology in more detail**

Within this review each domain was assessed based on the following scale: “no” (no risk of bias or imprecision or inconsistency or publication bias, depending on the domain: score 0), “serious” (serious risk of bias etc.: score -1) or “very serious” (very serious risk of bias or publication bias etc.: score -2). The basis of assessment was

- Risk of bias - Allocation concealment, lack of blinding and incomplete accounting for fate of participants. Serious risk of bias was indicated by poor allocation concealment, blinding or follow-up, or lack of reporting of any two of these elements. Very serious risk of bias was indicated by poor, or lack of reporting of, more than one of the elements of study validity. Selective outcome reporting is also an important element of study validity, but was omitted from this assessment as it is difficult to assess and would result in almost all validity assessments suggesting serious risk of bias. In observational studies confounding and similarity of the different groups at baseline needed to be assessed.
- Imprecision - serious imprecision was assumed to occur when the review collated <300 total events (dichotomous outcomes) or a total population size <400 (continuous outcomes). Very serious imprecision occurred when there were <100 total events or a population size of <150 or where these were not reported and could not be estimated. Where the events or population were not reported, but the effect was statistically significant the evidence were assessed as at serious risk of bias. Where the number of events was not provided for dichotomous outcomes then we assumed that imprecision was serious (rather than very serious) if the review stated that there were at least 1000 participants.
- Inconsistency - Unexplained heterogeneity of results. Scored “no” where heterogeneity was not present, or where it was present but explored, and scored “serious” where heterogeneity was clearly present (stated by the reviewers or clearly observable in the forest plot) but not explored or explained, or where not reported, or where only 1 study was found (as homogeneity could not be corroborated).
- Indirectness of evidence - presence of an indirect comparison or indirect evidence (studies did not directly address the question). Where intermediate markers such as entomological parameters were measured rather than health outcomes such as actual cases of disease or disease side effects such as low serum haemoglobin and miscarriage (side effects of malaria), this was considered indirect evidence (scored as serious risk of indirectness of evidence).
- Publication bias was assessed as “undetected” (score 0) or “strongly suspected” (score -1) on the basis that it was assessed and no evidence of bias was found. Where no information was presented this was scored “strongly suspected” unless there were fewer than 10 included studies as publication bias is difficult to assess in the presence of so few studies.

The GRADE summary score was the addition of the previous scores. These scores could be upgraded by the following factors: a large effect size (RR>2 or <0.5 score +1, RR>5 or <0.2 score +2), confounders working against bias (score +1), and/or presence of a dose response (score +1).
