# Supplementary material for: Public Health Interventions for Aedes Control in the Time of Zikavirus– A Meta-Review on Effectiveness of Vector Control Strategies
Source: PLoS Negl Trop Dis. 2016 Dec 7;10(12):e0005176. doi: 10.1371/journal.pntd.0005176 (PMC5142773; doi:10.1371/journal.pntd.0005176)
Supplement: S1 Text — (DOCX) [file pntd.0005176.s003.docx]

S1_Text. Emerging evidence about *Aedes* or *Aedes*-disease control not included in systematic reviews

Systematic reviews tend to provide dated information. The latest search date for any of our included systematic reviews was January 2015 ^1^. This means that articles published after the final search date on any individual review question will not inform our synthesis. To briefly consider the evidence base not assessed in published systematic reviews, the authors searched PubMed for either ‘mosquito’ or ‘dengue’ among the title, abstract or keywords of articles published 2014-2016. Single researcher screening of the results found sixteen primary research articles about community based studies to control *Aedes* vectors or dengue, which were not included in any of our eligible systematic reviews ^2-17^. The data in these articles can only be thoroughly assessed after full validity analysis and alongside any similar intervention reports, so their findings are only summarised briefly here. Four studies were clustered RCTs ^2,3,6,11^, four articles had contemporary comparators ^8,9,12,13^, and the other eight studies made pre-post comparisons in trying to evaluate impact of interventions. Within this group, only one study (a clustered RCT) ^3^ relied on routine intensive monitoring and regular application of insecticides and larvicides: the authors concluded that “In spite of extensive implementation of all standard control actions recommended by the Brazilian dengue control programme, only a slight decrease in mosquito density was detected”. A Cuban clustered RCT ^6^ had similar results, finding that use of insecticide treated curtains in an area with established routine *Aedes* control measures did not further reduce infestation levels.

The intervention models in these unsynthesised studies utilised biological agents ^4,13-16^, community education and mobilisation ^2,3^, environmental application of chemical control agents ^3,7,10-12,17^, baited traps (combined with a control agent or mechanism) ^5,8,9^ or nets or screens ^3,6,11^. The most effective clustered RCT intervention was based purely on social participation measures (no insecticide, biological or other measures were included with the intervention) ^2^. This Brazilian study reported that four vector indices were statistically significantly reduced when comparing control and intervention areas, in the rainy season. The remaining clustered RCT ^11^, that was not included in any systematic review, found that installation of insecticide treated curtains combined with provision of insecticide-treated water container covers led to a significant (p=0.01) reduction in Aedes aegypti pupae per person (71% to 25%), but observations were only made for a short period (six months).

There are novel aspects in some of the most recent research that may be desirable for a future systematic review to focus upon. Marini and colleagues ^17^ and Englbrecht and colleagues ^9^ provided the only studies about *Aedes* control in Europe. Kumar and colleagues ^14^ was the only study to report on the effects on *Aedes* larvae of an introduced beetle predator. In addition, two studies reported on field trials of the release of *Wolbachia* infected *Aedes* mosquitoes that resulted in lowering mosquito populations and reducing dengue transmission ^4,15^. Because insecticide resistance is an ongoing challenge for vector control ^18^, it is likely that interest in alternative interventions will continue to be high. We are aware of novel sustainable approaches not yet tested in field trials ^19,20^.

1. Bowman LR, Donegan S, McCall PJ. Is Dengue Vector Control Deficient in Effectiveness or Evidence?: Systematic Review and Meta-analysis. *PLoS neglected tropical diseases* 2016; **10**(3): e0004551.

2. Caprara A, Lima JWDO, Peixoto ACR, et al. Entomological impact and social participation in dengue control: a cluster randomized trial in Fortaleza, Brazil. *Transactions of the Royal Society of Tropical Medicine and Hygiene* 2015; **109**(2): 99-105.

3. Maciel-de-Freitas R, Valle D. Challenges encountered using standard vector control measures for dengue in Boa Vista, Brazil. *Bulletin of the World Health Organization* 2014; **92**(9): 685-9.

4. Nguyen TH, Le Nguyen H, Nguyen TY, et al. Field evaluation of the establishment potential of wmelpop Wolbachia in Australia and Vietnam for dengue control. *Parasites & vectors* 2015; **8**(1): 1-14.

5. Revay EE, Müller GC, Qualls WA, et al. Control of Aedes albopictus with attractive toxic sugar baits (ATSB) and potential impact on non-target organisms in St. Augustine, Florida. *Parasitology research* 2014; **113**(1): 73-9.

6. Toledo ME, Vanlerberghe V, Lambert I, Montada D, Baly A, Van der Stuyft P. No effect of insecticide treated curtain deployment on Aedes infestation in a cluster randomized trial in a setting of low dengue transmission in Guantanamo, Cuba. *PloS one* 2015; **10**(3): e0119373.

7. Caputo B, Manica M, D’Alessandro A, et al. Assessment of the Effectiveness of a Seasonal-Long Insecticide-Based Control Strategy against Aedes albopictus Nuisance in an Urban Area. *PLoS neglected tropical diseases* 2016; **10**(3): e0004463.

8. Nagpal B, Ghosh S, Eapen A, et al. Control of Aedes aegypti and Ae. albopictus, the vectors of dengue and chikungunya, by using pheromone C21 with an insect growth regulator: Results of multicentric trials from 2007-12 in India. *Journal of Vector Borne Diseases* 2015; **52**(3): 224.

9. Englbrecht C, Gordon S, Venturelli C, Rose A, Geier M. Evaluation of BG-Sentinel trap as a management tool to reduce Aedes albopictus nuisance in an urban environment in Italy. *Journal of the American Mosquito Control Association* 2015; **31**(1): 16-25.

10. Abad-Franch F, Zamora-Perea E, Ferraz G, Padilla-Torres SD, Luz SL. Mosquito-disseminated pyriproxyfen yields high breeding-site coverage and boosts juvenile mosquito mortality at the neighborhood scale. *PLoS neglected tropical diseases* 2015; **9**(4): e0003702.

11. Quintero J, García-Betancourt T, Cortés S, et al. Effectiveness and feasibility of long-lasting insecticide-treated curtains and water container covers for dengue vector control in Colombia: a cluster randomised trial. *Transactions of the Royal Society of Tropical Medicine and Hygiene* 2015; **109**(2): 116-25.

12. Harris JW, Richards SL, Anderson A. Emergency Mosquito Control on a Selected Area in Eastern North Carolina After Hurricane Irene. *Environmental health insights* 2014; **8**(Suppl 2): 29.

13. Alarcón ÉP, Segura ÁM, Rúa-Uribe G, Parra-Henao G. Ovitraps evaluation for surveillance and control of Aedes aegypti in two urban settlements of Urabá, Antioquia. *Biomédica* 2014; **34**(3): 409-24.

14. Kumar N, Bashir A, Abidha S, Sabesan S, Jambulingam P. Predatory potential of Platynectes sp.(Coleoptera: Dytiscidae) on Aedes albopictus, the vector of dengue/chikungunya in Kerala, India. *Tropical biomedicine* 2014; **31**(4): 736-41.

15. Hoffmann AA, Iturbe-Ormaetxe I, Callahan AG, et al. Stability of the w Mel Wolbachia infection following invasion into Aedes aegypti populations. *PLoS neglected tropical diseases* 2014; **8**(9): e3115.

16. Williams GM, Faraji A, Unlu I, et al. Area-wide ground applications of Bacillus thuringiensis var. israelensis for the control of Aedes albopictus in residential neighborhoods: from optimization to operation. *PloS one* 2014; **9**(10): e110035.

17. Marini L, Baseggio A, Drago A, et al. Efficacy of Two Common Methods of Application of Residual Insecticide for Controlling the Asian Tiger Mosquito, Aedes albopictus (Skuse), in Urban Areas. *PloS one* 2015; **10**(8): e0134831.

18. Macoris MdLdG, Andrighetti MTM, Wanderley DMV, Ribolla PEM. Impact of insecticide resistance on the field control of Aedes aegypti in the State of São Paulo. *Revista da Sociedade Brasileira de Medicina Tropical* 2014; **47**(5): 573-8.

19. Ramirez JL, Short SM, Bahia AC, et al. Chromobacterium Csp_P reduces malaria and dengue infection in vector mosquitoes and has entomopathogenic and in vitro anti-pathogen activities. *PLoS Pathog* 2014; **10**(10): e1004398.

20. Qureshi N, Chawla S, Likitvivatanavong S, Lee HL, Gill SS. The Cry Toxin Operon of Clostridium bifermentans subsp. malaysia Is Highly Toxic to Aedes Larval Mosquitoes. *Applied and environmental microbiology* 2014; **80**(18): 5689-97.
